# Downregulation of miR-432 activates Wnt/β-catenin signaling and promotes human hepatocellular carcinoma proliferation

**DOI:** 10.18632/oncotarget.3492

**Published:** 2015-03-08

**Authors:** Nan Jiang, Wen-Jie Chen, Jian-Wen Zhang, Chi Xu, Xian-Cheng Zeng, Tong Zhang, Yang Li, Guo-Ying Wang

**Affiliations:** ^1^ Department of Hepatic Surgery, The Third Affiliated Hospital of Sun Yat-Sen University, Guangzhou, Guangdong, China; ^2^ Department of General Surgery and Clinical Laboratory, Zengcheng People's Hospital, (BoJi-Affiliated Hospital of Sun Yat-Sen University), Zengcheng, Guangdong, China

**Keywords:** MiR-432, Wnt/β-catenin, Proliferation, Hepatocellular carcinoma

## Abstract

Sustained cell growth and proliferation, one of the hallmarks of cancer, is considered to responsible for cancer-related deaths by disorganizing the balance of growth promotion and growth limitation. Aberrant activation of the Wnt/β-catenin signaling pathway leads to cell proliferation, growth and survival, and promotes the development of various human tumors, including hepatocellular carcinoma. Elucidating the molecular mechanism of this abnormality in hepatocellular carcinoma carcinogenesis may improve diagnostic and therapeutic strategies for this malignancy. Herein, we report that the expression of miR-432 was markedly downregulated in hepatocellular carcinoma cell lines and tissues, and upregulation of miR-432 inhibited, whereas downregulation of miR-432 enhanced the proliferation and tumorigenicity of hepatocellular carcinoma cells both *in vitro* and *in vivo*. Furthermore, miR-432 directly targeted and suppressed multiple regulators of the Wnt/β-catenin signaling cascade, including LRP6, TRIM29 and Pygo2, which subsequently deactivated Wnt/β-catenin signaling pathway. Finally, miR-432 expression was inversely correlated with three targets in clinical hepatocellular carcinoma samples. These results demonstrated that miR-432 functions as a tumor-suppressive miRNA by suppressing Wnt/β-catenin signaling activation and may represent a therapeutic target for hepatocellular carcinoma.

## INTRODUCTION

Hepatocellular carcinoma (HCC) is the third leading cause of cancer mortality in the world and is associated with poor survival [1-3]. The incidence of HCC has been increasing dramatically over the last 20 years, particularly associated with hepatitis B or C virus (HBV or HCV) infection [1, 4]. Until now, the main curative treatment for HCC patients is surgical hepatic resection and liver transplantation. However, the therapeutic effect and prognosis for HCC patients has remained dismal, with survival rates of 20-65% at 1 year, 10-30% at 3 years and 10-20% at 5 years [5]. Therefore, identifying effective treatment strategies and understanding the molecular mechanisms responsible for the pathogenesis of HCC are needed urgently.

The Wnt/β-catenin pathway is an important regulator of tumor initiation and progression. Hyperactivation of this pathway promotes cell proliferation, survival and appears to control characteristics of the malignant phenotype in various cancers, including HCC [6]. Constitutive activation of Wnt/β-catenin signaling in cancer cells is achieved mainly by loss-of-function mutations in the APC (adenomatous polyposis coli) or Axin genes (axis inhibitor), or by activating mutations in the N-terminal domain of β-catenin [7]. Approximately 20% of HCCs have mutations in the β-catenin gene [8, 9], whereas APC and AXIN mutations are less frequent [9, 10]. However, β-catenin nuclear accumulation has been observed in more than 50% of HCC tumors [11-13], which suggested that there are other mechanisms involved in activation of the β-catenin signaling pathway in HCC. Abnormal expression of several components, such as WNTs, LRPs, TRIM29 and Pygopus, contribute to the activation of the Wnt/β-catenin pathway [14-17]. For instance, overexpression of LRP6, a member of the low-density lipoprotein (LDL) receptor family, caused hyperactivation of the Wnt/β-catenin signaling pathway in human HCCs and may play an important role in hepatocarcinogenesis [18]. Immunohistochemical analysis showed that the expression of TRIM29 is increased in pancreatic cancers and correlates with high expression levels of β-catenin [17]. Kramps *et al.* reported that the recruitment of Pygopus is regulated by BCL9 and promotes nuclear accumulation of β-catenin and transcriptionally activate Wnt target genes in B cell malignancies [19]. Thus far, the molecular mechanisms by which these components contributed to the development and progression of HCC remain incompletely understood. Therefore, a systemic understanding of the molecular mechanism of these regulatory effects on the Wnt/β-catenin signaling pathway is both biologically and clinically important for future HCC research and therapy.

MicroRNAs (miRNAs), a class of endogenous noncoding small RNAs, negatively regulate target gene expression and are involved in the modulation of many biological processes [20]. Accumulating evidence demonstrated that miRNAs play an important role in the initiation and progression of human cancers and, therefore, may represent promising targets for anticancer therapies [21, 22]. One miRNA usually targets multiple mRNAs of different genes, therefore, it is of particular interest to identify those miRNAs that can simultaneously interact with multiple regulators of the β-catenin pathway and thereby lead to cancer proliferation. Herein, we report that miR-432 deactivates the Wnt/β-catenin pathway by simultaneously suppressing the expression of LRP6, TRIM29, and Pygo2, and consequently repress proliferation in HCC.

## RESULTS

### MiR-432 is downregulated in HCC

**Fig.1:**
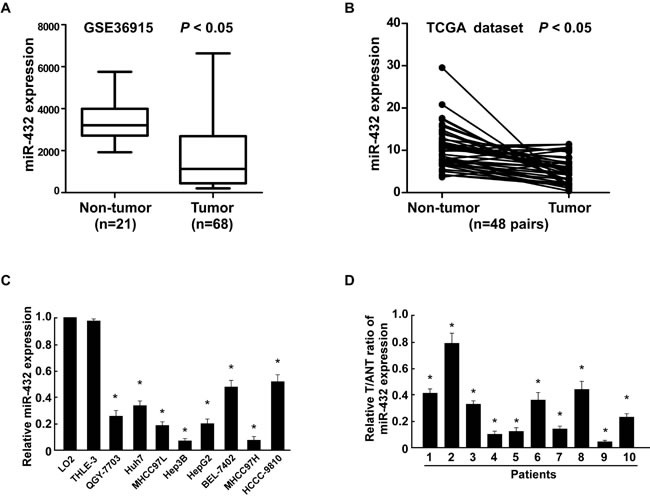
MiR-432 is downregulated in HCC cell lines and tissues (A). Expression profiling of miRNAs showing that miR-432 is downregulated in HCC tissues (T) compared with matched non-cancerous liver tissue (N) (n = 89, p<0.001; NCBI /GEO /GSE 36915). (B). Expression profiling of miR-432 from The Cancer Genome Altas (TCGA) datasets in liver hepatocellular carcinoma ((http://cancergenome.nih.gov/). (C). Real-time PCR analysis of miR-432 expression in normal human LO2 hepatocyte and in HCC cell lines (Hep3B, MHCC97L, Huh7, HCCC-9810, HepG2, BEL-7402, MHCC97H and QGY-7703). Transcript levels were normalized to *U6* expression. (D). Real-time PCR analysis of miR-432 expression in primary HCC tissues (T) with matched adjacent non-tumor tissues (ANT) from eight individual patients. Transcript levels were normalized to *U6* expression. Each bar represents the mean ± SD of three independent experiments. *P < 0.05.

By analyzing a published microarray-based high-throughput assessment, miR-432 was identified to be significantly downregulated in HCC tissues compared with non-cancerous liver tissue (n = 89; *P* < 0.05; NCBI/GEO/GSE36915; Figure 1A). Consistent with this observation, we further analyzed the data from The Cancer Genome Atlas (TCGA) (http://cancergenome.nih.gov/, n=48 pairs) in liver hepatocellular carcinoma and matched non-cancerous liver tissue and found that miR-432 was significantly downregulated in tumor samples (Figure 1B). Strikingly, real-time PCR showed that miR-432 was downregulated in all 10 tumor tissue samples and in eight HCC cell lines (QGY-7703, Huh7, MHCC97L, Hep3B, HepG2, BEL-7402, MHCC97H, HCCC-9810)compared with adjacent non-cancerous tissues and normal human LO2 hepatocyte, respectively (Figure 1C and 1D). Collectively, these results indicated that miR-432 is downregulated in HCC.

### Ectopic expression of miR-432 inhibited HCC cell proliferation *in vitro*

**Fig.2:**
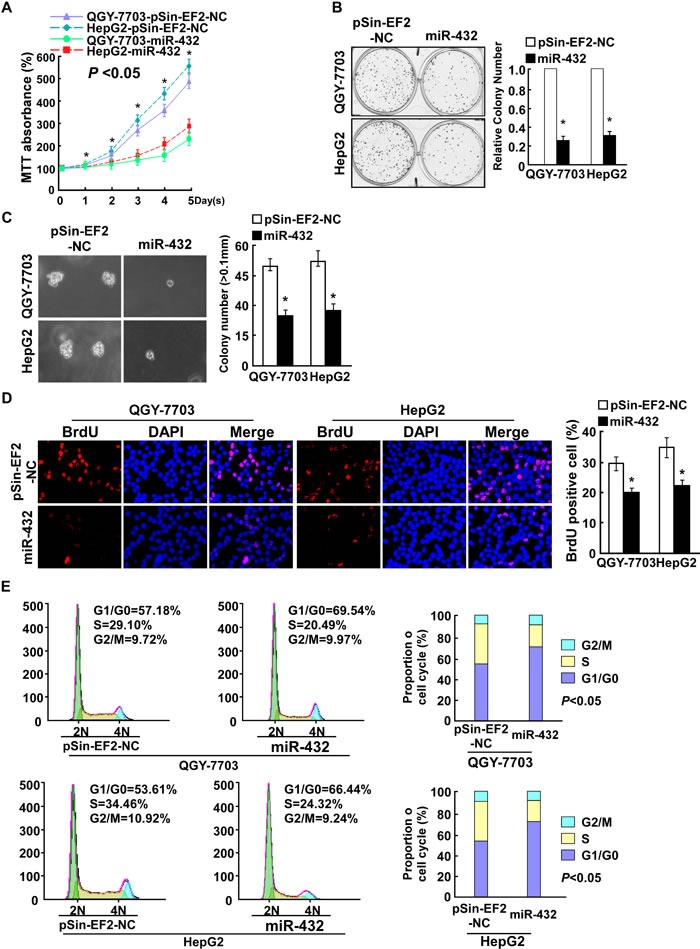
Ectopic expression of miR-432 inhibited HCC cell proliferation (A). MTT assay revealed that miR-432 upregulation suppresses QGY-7703 and HepG2 stable cell lines at indicated times after seeding. (B). Representative micrographs (left) and quantification (right) of crystal violet-stained cell colonies. Indicated cells (0.5 × 10^3^) were plated into six-well plates and cultured for 10 days, then stained with crystal violet (1.0%). (C). Representative micrographs (left) and quantification of colonies > 0.1 mm (right) were scored. Indicated cells (2 × 10^3^) were suspended in soft agar and cultured for 10 days, and then colonies > 0.1 mm in diameter were counted. (D). Representative micrographs (left) and quantification of BrdU positive signaling in the cells transfected with miR-432 or Vector. (E). Flow cytometry analysis of indicated HCC cells stably expressing miR-432 or Vector. Each bar represents the mean ± SD of three independent experiments. *P < 0.05.

To determine the effect of miR-432 on HCC progression, QGY-7703 and HepG2 cells were selected for further study (Supplemental Figure 1). As shown in Figure 2A and 2B, upregulation of miR-432 significantly decreased the growth rate of QGY-7703 and HepG2 cells, analyzed by MTT and colony formation assays. Importantly, the anchorage-independent growth ability of QGY-7703 and HepG2 cells was drastically repressed upon miR-432 overexpression cells, as indicated by the reduction in colony number and colony size on soft agar (Figure 2C), suggesting that miR-432 upregulation decreased the tumorigenicity of HCC cells *in vitro*.

Furthermore, BrdU incorporation assay showed that the percentage of cells in S phase was dramatically decreased in miR-432-overexpressing HCC cells compared with the control cells (Figure 2D). Similarly, flow cytometry showed that miR-432 overexpression decreased the percentage of HCC cells in S phase and significantly increased the percentage of cells in G1/G0 (Figure 2E). Collectively, our results suggested that miR-432 upregulation inhibits HCC cell proliferative capacity *in vitro* through regulation of G1/S transition.

### MiR-432 inhibition promoted HCC cell proliferation *in vitro*

**Fig.3:**
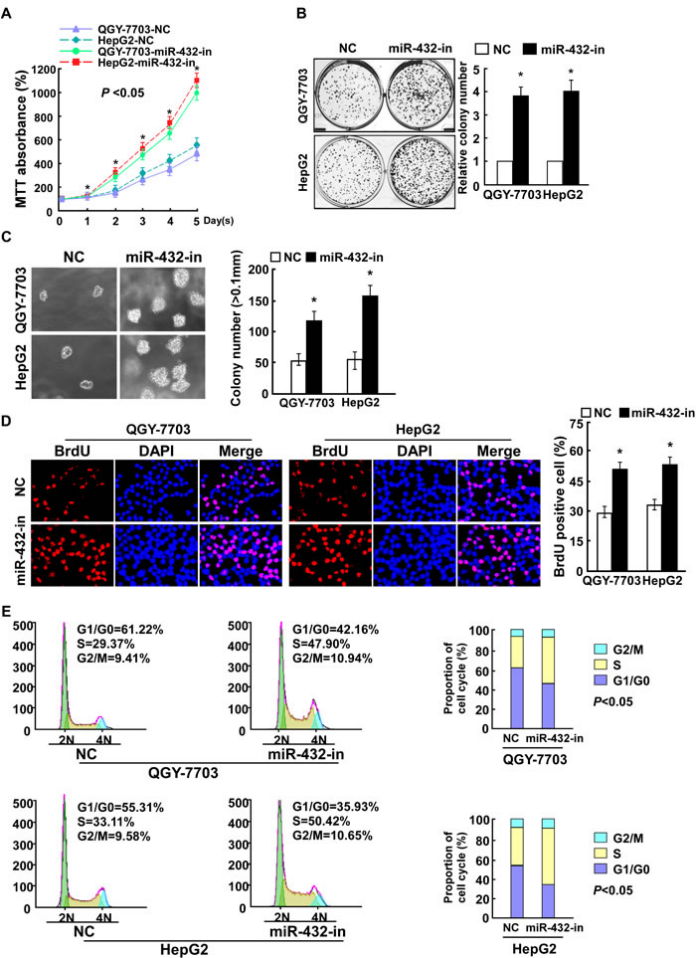
Inhibiting miR-432 expression enhanced HCC cell proliferation (A). MTT analysis of the proliferation ability of HCC cells transfected with miR-432-in or NC. (B). Representative micrographs (left) and quantification (right) of HCC cell colonies in indicated HCC cell lines, as determined by colony formation assay. (C). Tumorigenicity of HCC cells transfected with miR-432-in or negative control (NC) was measured by anchorage-independent growth ability assay. Colonies larger than 0.1 mm in diameter were scored. (D). Representative micrographs (left) and quantification of BrdU positive signaling in the cells transfected with miR-432-in or NC. (E). Flow cytometry analysis of indicated HCC cells transfected with miR-432-in or NC. Each bar represents the mean ± SD of three independent experiments. * *P* < 0.05.

We further examined the effect of miR-432 inhibition on HCC cell proliferation. Consistent with the overexpression results, MTT and colony formation assays showed that miR-432 suppression dramatically increased the growth rate of both miR-432 overexpression cells compared with that of control cells transfected with negative control (NC) (Supplemental Figure 1, Figure 3A and 3B). In addition, the anchorage-independent growth ability of QGY-7703 and HepG2 HCC cells was significantly increased in response to miR-432 inhibitor (Figure 3C). Furthermore, we found that transfection of miR-432 inhibitor drastically increased the percentage of cells in the S peak but decreased that in the G0/G1 peak (Figure 3D and 3E). Taken together, these results suggest that miR-432 downregulation promotes the proliferation of HCC cells *in vitro*.

### MiR-432 downregulation contributed to HCC progression *in vivo*

**Fig.4:**
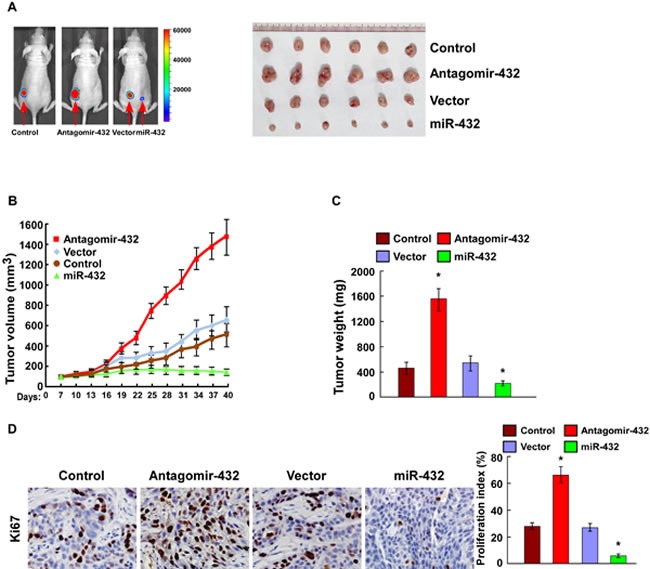
MiR-432 downregulation contributed to HCC progression *in vivo* (A). Representative images of tumor-bearing mice (left) and images of the tumors from all of the mice in each group (right). (B). Tumor volumes were measured on the indicated days. (C). Mean tumor weights. (D). IHC staining showing that miR-432 downregulation increased the percentages of Ki-67 positive cells, whereas miR-432 overexpression inhibited the percentages of Ki-67 positive cells. Each bar represents the mean ± SD of three independent experiments. * *P* < 0.05.

To investigate whether miR-432 inhibits HCC progression *in vivo*, we used a xenografted tumor model to examine the biological effect of miR-432. As shown in Figure 4A-4C, the tumors formed by miR-432-silenced cells were larger in both size and weight than the tumors formed by control cells. Conversely, the tumors formed by miR-432-transduced-HCC cells were smaller and lighter than the control tumors. Furthermore, IHC analysis revealed that miR-432-silenced tumors showed higher percentages of Ki-67-positive cells, whereas miR-432-overexpressing tumors displayed lower percentages of Ki-67 positive cells than the control tumors (Figure 4D). Collectively, these results demonstrate that miR-432 finctions as a tumor suppressor in HCC *in vivo*.

### MiR-432 suppressed LRP6, TRIM29, and Pygo2 directly

**Fig.5:**
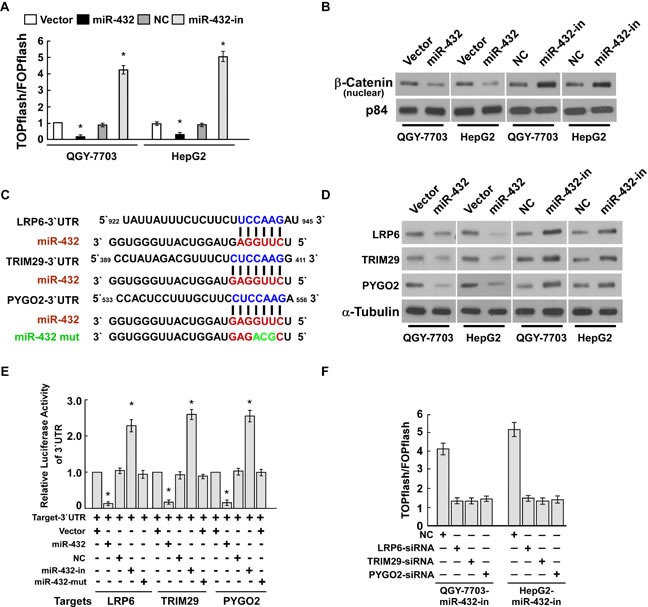
LRP6, TRIM29 and Pygo2 are direct targets of miR-432 (A). Indicated cells transfected with TOPflash or FOPflash and Renilla pRL-TK plasmids were subjected to dual-luciferase assays 48 hours after transfection. Reporter activity detected was normalized by Renilla luciferase activity. (B). Altered nuclear translocation of β-catenin in response to deregulated miR-432 expression. Nuclear fractions of indicated cells were analyzed by western blotting; p84 was used as the loading control. (C). Predicted miR-432 target sequences in the 3′UTRs of LRP6, TRIM29 and Pygo2. The miR-432 mutant (miR-432-mut) contained three altered nucleotides in the seed sequence. (D). Western blotting analysis of LRP6, TRIM29 and Pygo2 expression in the indicated HCC cells. α-Tubulin served as loading control. (E). Luciferase assay of pGL3- *LRP6*-3′UTR, pGL3- *TRIM29*-3′UTR and pGL3- *Pygo2*-3′UTR reporter cotransfected with miR-432, miR-432-inhibitor and miR-432-mut oligonucleotides in HCC cells. (F). Luciferase assay of TCF/LEF transcriptional activity in indicated cells transfected with specific siRNA. Error bars represent the mean ± SD from of three independent experiments. *P < 0.05.

The wnt/β-catenin pathway is an important regulator of tumor initiation and progression, and palys an important role in cell proliferation. Therefore, we detect the effect of miR-432 in regulating the wnt/β-catenin signaling. As shown in Figure 5A, miR-432 overexpression markedly decreased the luciferase activity of TOPflash or FOPflash reporter; conversely, transfection of miR-432 inhibitor increased the luciferase activity of TOPflash or FOPflash reporter, compare with vector or negative control, respectively. Furthermore, cellular fractionation showed that miR-432 overexpression inhibit nuclear accumulation of β-catenin (Figure 5B), indicating that miR-432 deactivates Wnt/β-catenin pathway by inhibiting β-catenin nuclear accumulation.

To explore the mechanism underlying the suppressive effect of miR-432 on Wnt/β-catenin signaling, we used publicly available algorithms (TargetScan and miRanda) to predict the potential targets of miR-432 in humans. Figure 5C shows that three critical components of the Wnt/β-catenin signal pathway, including LRP6, TRIM29, and Pygo2, are potential targets of miR-432. As expected, western blotting showed that LRP6, TRIM29, and Pygo2 expression in both QGY-7703 and HepG2 cells were dramatically downregulated in response to miR-432 transfection, and upregulated by the miR-432 inhibitor (Figure 5D).

We further performed luciferase reporter assay to confirm whether LRP6, TRIM29, and Pygo2 are direct targets of miR-432. As shown in Figure 5E, ectopic expression of miR-432 decreased the luciferase activities of the 3′UTRs of LRP6, TRIM29, and Pygo2. By contrast, transfection with the miR-432 inhibitor increased the luciferase activities. However, a miR-432 mutant containing three altered nucleotides in the seed sequence showed no inhibitory effect on luciferase activity. Individually silencing of LRP6, TRIM29 or Pygo2 decreased the transcriptional activity of the downstream effectors TCF and LEF in HCC cells whose miR-432 expression was suppressed (Figure 5F). These results demonstrated that miR-432 suppressed LRP6, TRIM29, and Pygo2 directly in HCC cells.

### MiR-432 overexpression inhibited the Wnt/β-catenin signaling pathway

The above mentioned miR-432 targets are closely correlated with Wnt/β-catenin signaling pathway, therefore, we further examined the effect of miR-432 deregulation on the expression of downstream genes of the Wnt/β-catenin signaling pathway. Supplemental Figure 2 illustrates that the mRNA level of β-catenin downstream targets, including Cyclin D1, MYC, TCF4 and LEF1, were significantly decreased in miR-432-overexpressing cells, whereas their mRNA levels were increased in cells transfected with the miR-432 inhibitor. Taken together, our results suggest that miR-432 overexpression inhibits the Wnt/β-catenin signaling pathway.

### Suppression of LRP6, TRIM29, and Pygo2 is functionally important for the biological effects of miR-432 in HCC

To examine the effect of LRP6, TRIM29, and Pygo2 suppression on the proliferative capacity of the miR-432 inhibitor in HCC cells, we studied the effects of their depletion using specific siRNAs. As shown in Supplemental Figure 3A-3D, individual silencing of these target genes decreased cell growth rate and proliferation potently, as assessed by colony formatio, anchorage-independent growth and BrdU incorporation assays, respectively. These results suggest that LRP6, TRIM29, and Pygo2 are essential for miR-432 downregulation-mediated HCC cell proliferation.

### Clinical relevance of miR-432, β-catenin nuclear accumulation and expression of its targets in HCC

**Fig.6:**
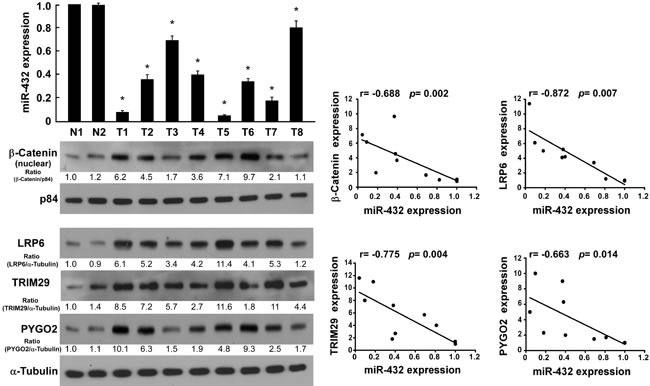
Clinical relevance of miR-432 downregulation and β-catenin nuclear accumulation, and the expression of LRP6, TRIM29 and Pygo2 in HCC (A). Expression of miR-432 and β-catenin nuclear accumulation(p84 served as loading control), LRP6, TRIM29 and Pygo2 protein, as measured by real-time PCR (top) and western blotting (bottom), respectively. α-Tubulin served as loading control. (B). Correlation between miR-432 and β-catenin LRP6, TRIM29 and Pygo2 expression was analyzed by SPSS software. Error bars represent the mean ± SD from of three independent experiments. *P < 0.05.

Finally, we examine whether miR-432-mediated suppression of LRP6, TRIM29, and Pygo2 and β-catenin nuclear accumulation in HCC are clinically relevant. Using 10 freshly collected clinical HCC samples, we found that miR-432 expression was inversely correlated with the expression of β-catenin(r = −0.688, *P* = 0.002), LRP6 (r = −0.872, *P* = 0.007), TRIM29 (r = −0.775, *P* = 0.004), Pygo2 (r = −0.663, *P* = 0.014) (Figure 6A and 6B). Collectively, these results support the notion that miR-432 downregulation promotes proliferation in HCC and activates the Wnt/β-catenin signaling pathway by repressing multiple important regulator this pathway.

## DISCUSSION

Herein we provide evidence for a novel mechanistic link between miR-432 and the oncogenic Wnt/β-catenin activity in HCC. MiR-432 expression is markedly downregulated in HCC cells and clinical tissues. Ectopic expression of miR-432 inhibited, whereas repression of endogenous miR-432 promoted the proliferation and tumorigenicity of HCC cells *in vitro* and *in vivo*. Furthermore, we demonstrated that miR-432 attenuated the activity of Wnt/β-catenin signaling, from plasma membrane to nucleus levels, by suppressing the expression of LRP6, TRIM29, and Pygo2 in HCC, which suggested that miR-432 acts as a tumor suppressor, and may represent an important target for clinical intervention in HCC by controlling Wnt/β-catenin signaling.

MicroRNAs have been demonstrated to negatively regulate target mRNAs in a sequence-specific manner, and are key regulators in a wide variety of oncogenic processes, such as cell proliferation, differentiation, invasion and metastasis, functioning as either tumor suppressors or oncogenes [20, 22, 23]. Therefore, elucidating the underlying mechanism of miRNAs in tumor development may provide valuable diagnostic and therapeutic strategies for malignancy. MiR-432 was downregulated in multiple tumors, for instance, ovarian cancer, cervical cancer, human pituitary GH adenomas, and plays a role as tumor suppressor gene by different mechanism [24-26]. A newly study reveal that miR-432 induced neurite projections, arrested cells in G0–G1, reduced cell proliferation and could signiﬁcantly repress the expression of NESTIN/NES, RCOR1/COREST and MECP2,which suggest that miR-432 may play an important role in regulating neuronal differentiation of human neuroblastoma cells [27]. Interestingly, we also found that mir-432 expression was markedly downregulated in HCC cells and tissues and overexpression of miR-432 inhibited the proliferation and tumorigenicity of HCC cells *in vitro* and *in vivo*. Furthermore, we demonstrated that miR-432 attenuated the activity of Wnt/β-catenin signaling by suppressing the expression of LRP6, TRIM29, and Pygo2 in HCC, which suggesting that miR-432 act as a tumor suppressor and may represent a new target for clinical intervention of HCC by controlling Wnt/β-catenin signaling. Consistent with previous studies, our studies demonstrate an important role of miR-432 in modulating tumor development and may represent a promising therapeutic target in HCC. However, there are other studies showed that miR-432 was amplified in metastatic melanoma and upregulation of miR-432 markedly enhanced the tumorigenicity *in vivo* by modulating ADAR1 expression [28]. Thus, the above findings suggest that miR-432 expression and its biological functions might be tumor-type dependent. The clinical significance and mechanism of miR-432 downregulation in HCC require further investigation.

Deregulation of the Wnt/β-catenin signaling pathway is common in many types of cancer and activation of this pathway is thought to be an early event in tumorigenesis [7, 29-33]. Mutations in the β-catenin gene are observed in only 12–26% of HCCs, but aberrant cytoplasmic and nuclear accumulation of β-catenin are more common, occurring in 40–70% of HCCs [9-13, 34], suggesting that there are other mechanisms involved in activation of the β-catenin signaling pathway in HCC. Thus, understanding whether and how a specific factor in HCC cells can simultaneously interact with multiple regulators of β-catenin signaling may provide new insights into the molecular mechanisms underlying cancer development. Our current study shows that miR-432 simultaneously represses the expression of three important factors of β-catenin pathway: LRP6, TRIM29, and Pygo2, which subsequently suppresses β-catenin activation in HCC. These three identified mediation molecules have all been suggested to be upregulated in multiple tumors and play important roles in the modulation of oncogenic β-catenin signaling from the plasma membrane to nucleus levels. First of all, most data show that LRP6 functions as an indispensable membranous co-receptor for the Wnt signaling pathway by transmitting the extracellular signals to downstream factors, subsequently increasing β-catenin nuclear accumulation [35, 36]. LRP6 was reported to be upregulated in human cancers, including lung tumor, colon tumor, HCC and to promote the progression of tumors through regulation of the Wnt/β-catenin signaling pathway [15]). Secondly, TRIM29, a member of tripartite motif (TRIM) family, is located on chromosome 11q23 and was reported to be upregulated in multiple types of cancers including pancreatic, gastric, bladder, ovarian and colorectal [17, 37-40]. Wang *et al.* demonstrated that high expression of TRIM29 correlated with high β-catenin levels in pancreatic cancer and that is promoted cancer cell proliferation *in vitro* and enhanced tumor growth and metastasis *in vivo* [17]. Importantly, Wang revealed the mechanism by which ATDC stabilizes β-catenin: in pancreatic cancer cells with high levels of ATDC, ATDC binds to and stabilizes Dvl-2 in the cytoplasm, resulting in the release of β-catenin from the destruction complex, which leads to increased β-catenin levels and activation of downstream β-catenin/TCF-regulated target genes. A new nuclear component of the Wnt signaling pathway, Pygo2, was upregulated in several tumors and is required for growth of epithelial ovarian cancer [41] and breast cancer [42]. Wang *et al*. demonstrated that Pygo2 was highly expressed in glioma tissue and required for growth of glioblastoma cells, however, knockdown of the Pygo2 expression by Pygo2 shRNA effectively suppressed malignant glioma cell proliferation, which was associated with cell cycle arrest at the G1 stage [43]. Thomas *et al.* revealed that Pygo2 is physically linkd to the β-catenin-TCF complex and that this recruitment of Pygo2 is required for β-catenin to function as a transcriptional coactivator [19]. Comprehensive gene expression profiling also showed that Pygo2 was upregulated in amplified chromosome regions in HCC, which suggested that Pygo2 may play an important role during HCC progression [44]. The regulatory networks that control such molecular alterations, however, have not been well defined in HCC. Herein, we demonstrate that miR-432 simultaneously suppresses three important component of the wnt pathway, i.e. LRP6, TRIM29, and Pygo2 expression by directly targeting their 3′UTRs and that β-catenin nuclear accumulation could be robustly repressed by miR-432 overexpression in HCC. Consistent with the oncogenic effects of LRP6, TRIM29, and Pygo2 in HCC, miR-432 is downregulated in HCC, and its suppression dramatically promoted HCC cell proliferation both *in vitro* and *in vivo*. Taken together, our results represent a novel mechanism that, from plasma membrane to nucleus, miR-432 suppressed the β-catenin pathway activation by repressed the expression of LRP6, TRIM29, and Pygo2 in HCC, and a functionally and clinically relevant epigenetic mechanism of HCC pathogenesis.

In summary, the present study demonstrated the tumor-suppressive effect of miR-432 in HCC. MiR-432 upregulation drastically suppress HCC cell proliferation by inhibiting the expression of three key regulators of the Wnt/β-catenin signaling pathway, i.e. LRP6, TRIM29, and Pygo2. These findings uncover a novel molecular mechanism of hyperactivation of the Wnt/β-catenin pathway in HCC and may prove clinically useful for developing new therapeutic targets for HCC.

## MATERIALS AND METHODS

### Cell culture

HCC cell lines, QGY-7703, Huh7, MHCC97L, Hep3B, HepG2, BEL-7402, MHCC97H, HCCC-9810, purchased from the American Type Culture Collection (ATCC, Manassas, VA, USA), were maintained in Dulbecco's modified Eagle's medium (Invitrogen, Carlsbad, CA, USA) supplemented with 10% fetal bovine serum (Invitrogen), 100U/ml penicillin and 100μg/ml streptomycin (Invitrogen), within a humidified atmosphere containing 5% CO_2_ at 37°C. Normal liver epithelial cell LO2 was purchased from the Chinese Academy of Sciences Committee Type Culture Collection cell bank and was cultured under the conditions stated by the manufacturer. Normal liver epithelial cell THLE-3 were purchased from the American Type Culture Collection (ATCC, Manassas, VA) and cultured as suggested by the manufacturer.

### Cell line authentication

All cell lines were authenticated by short tandem repeat (STR) fingerprinting at Medicine Lab of Forensic Medicine Department of Sun Yat-sen University (Guangzhou, China) within twelve months of the study.

### Tissue specimens

This study was conducted on 10 pairs of snap-frozen HCC tumors and matched normal tissues from adjacent regions, which were diagnosed histopathologically at the Third Affiliated Hospital of Sun Yat-sen University from 2001 to 2012. The 10 pairs HCC tissues and the matched adjacent noncancerous tissues were frozen and stored in liquid nitrogen until required. The information of clinical characteristics of all 10 patients, including 1 clinical stage I, 4 clinical stage II, 4 clinical stage III, 1 clinical stage VI.

For the use of the clinical materials for research purposes, prior patient consent and approval from the Institutional Research Ethics Committee were obtained.

Analysis of miR-432 expression in HCC tissues compared with that in matched noncancerous hepatic tissues was using a published microarray-based high-throughput assessment (n = 89; P < 0.05; NCBI/GEO/GSE36915). The data were downloaded from NCBI and analyzed using SPSS13.0 software.

### RNA extraction and real-time quantitative PCR (qRT-PCR)

The TRIzol reagent (Invitrogen) was used to extract total cellular RNA, according to the manufacturer's protocol. Complementary DNAs were synthesized and Real-time PCR was performed using RT Real-Time^TM^ SYBR Green (Bio-Rad Laboratories, Berkeley, CA). Expression levels of genes were normalized to that of the housekeeping gene GAPDH as the control and calculated as 2^−[(Ct of *Cyclin D1, MYC, TCF4, LEF1*) - (Ct of *GAPDH*)]^, where Ct represents the threshold cycle for each transcript. The following primers were synthesized and used in this study: *Cyclin D1* forward: 5′-AACTAC CTGG A CCGCTTCCT-3′; *Cyclin D1* reverse: 5′-CCACTTGAGCTTGTTCACCA-3′; *MYC* forward: 5′-TCAAGAGGCGA AC ACACAAC-3′; *MYC* reverse: 5′-GGCCTTTTCATTGT TTTCCA-3′; *TCF4* forward:5-CCAACTTCTTT GGCAAGTGG-3′; *TCF4* reverse: 5-TCT CCATAGT TCCTGGACGG-3′; *LEF1* forward: 5′-CACTGTAAGTGATGAGGGGG-3′; *LEF1* reverse: 5′-TGGATCTCTTTCTCCACCC A-3′; GAPDH forward: 5′-GACTCATG ACCACAGTCC ATGC-3′; GAPDH reverse: 3′-AGAGGCAGGGATGATGTTCTG-5′.

Total miRNA from cultured cells and HCC tissue samples was extracted using the mirVana miRNA Isolation Kit (Ambion, Austin, TX, USA) according to the manufacturer's manual. The expression level of miR-432 was performed using miR-432-specific primer and probe (TaqMan MicroRNA Assay Kit, Applied Biosystems, Foster City, CA) on an ABI 7900 system (Applied Biosystems). The expression of miR-432 was defined based on the threshold cycle (Ct), and relative expression levels were calculated as 2^−[(Ct of miR-432) − (Ct of U6)]^ after normalization with reference to the quantification of *U6* small nuclear RNA expression. The expression of the miRNA was defined based on Ct, and relative expression levels were calculated as 2^−[(Ct of miR-432) − (Ct of U6)]^ after normalization with reference to the expression of small nuclear RNA U6.

### Plasmid, siRNA and transfection

To generate a mir-432 expression vector, approximately 250-bp genomic fragment up and downstream of the pre-mir-432 form was generated by PCR amplification from genomic DNA, and subcloned into the EcoRI and SpeI sites of the pSin-EF2-puro retroviral vector (Clontech Laboratories Inc., Mountain View, CA). The primers for amplifying miR-432 were as the following: miR-432-up: 5′-GCCGAATTCATTCGGAAGTACAGCGAGAT-3′; miR-432-dn: 3′-GCCACTAGTTACTGACTGGACGCCATCAC-5′. The non-targeting control microRNA (negative control mimic), which are designed computationally to have no perfect seed-sequence matches to the transcriptome, was subcloned into retroviral transfer plasmid pSin-EF2-puro retroviral vector to generate the plasmid pSin-EF2-NC. The regions of the human LRP6 3′UTR, from 839-2878, TRIM29 3′UTR, from 186-518, and Pygo2 3′UTR, from 328-603, were PCR-amplified from genomic DNA and cloned separately into the pGL3 luciferase reporter plasmid (Promega, Madison, WI), respectively. The primers used were: *LRP6*-3′UTR-up: 5′-GCCGGATCCAATATTTCTTCTAGCTCCATTCC CC-3′; *LRP6*-3′UTR-dn: 3′-GCCACTAGTAGCAAACAAAACTCCTTAAGTCACT-5′; *TRIM29*-3′UTR-up: 5′-GCCGGATCCCATTCAGACTCCTTTCCTGCCTTGT-3′; *TRIM29*-3′UTR-dn: 3′-GCCACTAGTAGAGAGCACGTAGGGC CAGTCCTCT-5′; *Pygo2*-3′UTR-up: 5′-GCCGAATTCGTGTCCCTCTGCTGATGATGGAT G-3′; *Pygo2*-3′UTR-dn: 3′-GCCGAATTCGTGTTCCCTCTGCTGATGATGGATG-5′;

The reporter plasmids containing wild-type (CCTTTGATC; TOPflash) or mutated (CCTTTGGCC; FOPflash) TCF/LEF DNA binding sites were purchased from Upstate Biotechnology. MiR-432 mimic and miR-432 inhibitor (a cholesterol-conjugated 2′-O-Me modified antisense oligonucleotide designed specifically to bind to and inhibit endogenous miR-432 molecule) were synthesized and purified by RiboBio (Guangzhou, Guangdong). For depletion of LRP6, TRIM29 or Pygo2, siRNA was synthesized and purified by RiboBio. The siRNA sequences were: LRP6 siRNA: CCGATGCAATGGAGATGCAAA; TRIM29 siRNA: CCACGTTGAGAAGATGTGCAA; Pygo2 siRNA: TGTCGGAGTGAGGTGAACGAT; Transfection of miRNA and siRNAs was performed using the Lipofectamine 2000 reagent (Invitrogen) according to the manufacturer's instruction.

### Generation of stably engineered cell lines

pSin-EF2-miR-432 and pSin-EF2-NC were co-transfected with the PMD2g and PSPAX packaging plasmid into 293FT cells using the standard calcium phosphate transfection method. Twenty-four hours after co-transfection, supernatants were collected and incubated with cells to be infected for 24 hours in the presence of polybrene (2.5 μg/ml). After infection, puromycin(1.5 μg/ml) was used to select stably transduced cells over a 10-day period.

### Luciferase assay

Cells were seeded in triplicate in 24-well plate and allowed to settle for twenty-four hours. One hundred nanograms of pGL3- *LRP6*-luciferase plasmid, pGL3- *TRIM29*-luciferase plasmid, or pGL3- *Pygo2*-luciferase plasmid, plus 5ng of pRL-TK renilla plasmid (Promega, Madison, WI) were transfected into HCC cells using the Lipofectamine 2000 reagent (Invitrogen), respectively, according to the manufacturer's instruction. Luciferase and control signals were measured at 48h after transfection using the Dual Luciferase Reporter Assay Kit (Promega), according to a protocol provided by the manufacturer. Three independent experiments were performed and the data were presented as the mean ± SD.

### Western blotting

Cellular proteins were prepared in sample buffer [62.5mM Tris-HCl (pH 6.8), 10% glycerol, 2% SDS] and heated for 10 min at 100°C. Equal quantities of protein were electrophoresed through a 10% SDS/polyacrylamide gel and transferred to a PVDF membrane (Millipore, Billerica, MA, USA). The membranes were incubated with anti-LRP6 (Cell Signaling Technology, Danvers, MA, USA), anti-TRIM29 (Santa Cruz Biotechnology, Inc., Santa Cruz, CA, USA), anti-Pygo2 (Santa Cruz Biotechnology, Inc., Santa Cruz, CA, USA), anti-β-catenin (Cell Signaling Technology, Danvers, MA, USA). Nuclear extracts were prepared using the Nuclear Extraction Kit (Active Motif), according to the manufacturer's instructions. The membranes were stripped and re-blotted with an anti-Tubulin monoclonal antibody (Sigma, Saint Louis, MO, USA) as loading control.

The signals of western blotting bands of Figure 6 were quantified by densitometry, which determined by comparing the ratio in non-cancerous liver tissue (N1), *i.e.* the ratioβ-catenin /p84, LRP6/α-tubulin, or TRIM29/α-tubulin, or Pygo2/α-tubulin in N1 was considered as 1.0. The relative expression of miR-432 in tumors were quantified by real-time PCR, which determined by comparing the miR-432 expression in N1 (*i.e.* the expression of miR-432 was considered as 1.0). The correlation analysis between β-catenin /p84, LRP6/α-tubulin, or TRIM29/α-tubulin, or Pygo2/α-tubulin ratio with miR-432 expression was performed by Student's 2-tailed t-test.

### 3-(4, 5-Dimethyl-2-thiazolyl)-2, 5-diphenyl-2H-tetrazolium bromide (MTT) assay

HCC cells, seeded on 96-well plates, were stained at the indicated time points with 100 μl sterile MTT dye (0.5 mg/ml; Sigma) for 4 h at 37°C, followed by removal of the culture medium and the addition of 150 μl of dimethyl sulfoxide (Sigma). Absorbance was measured at 570 nm, with 655 nm as the reference wavelength. All experiments were performed in triplicates.

### Colony formation assay

Cells plated onto six-well plates at 0.5 × 10^3^cells per well were cultured for 10 days. Colonies were then fixed with 10% formaldehyde for 10 min and stained for 10 min with 1.0% crystal violet. All experiments were performed in triplicates.

### Anchorage-independent growth ability assay

Cells were trypsinized, and 2 × 10^3^ cells were resuspended in 2 ml complete medium plus 0.33% agar (Sigma). The agar–cell mixture was plated on top of a bottom layer consisting of 0.66% agar in complete medium. After 10 days, colony size was measured using an ocular micrometer and colonies larger than 0.1 mm in diameter were counted. The experiment was performed three times for each cell line.

### Bromodeoxyuridine labeling and immunofluorescence

Cells grown on cover slips (Fisher, Pittsburgh, PA, USA) were incubated with bromodeoxyuridine (BrdU) for 1 h and then stained with an anti-BrdU antibody (Upstate, Temecula, CA) according to the manufacturer's instructions. Gray level images were acquired using a laser scanning microscope (Axioskop 2 plus; Carl Zeiss Co. Ltd, Jena, Germany).

### Flow cytometry analysis

Cells in a culture dish were harvested by trypsinization, washed in ice-cold PBS, and fixed in 75% ice-cold ethanol in phosphate buffered saline (PBS). The cells were then pelleted and resuspended in cold PBS. Bovine pancreatic RNAase (2 μg/ml, Sigma) was added and cells were incubated at 37°C for 30 min, followed by incubation in propidium iodide (10 μg/ml, Invitrogen) for 30 min at 37°C. Twenty thousand cells were analyzed by flow cytometry (FACSCalibur, BD Biosciences, San Jose, CA). All experiments were performed in triplicates.

### Xenografted tumor model and immunohistochemistry(IHC)

All experimental procedures were approved by the Institutional Animal Care and Use Committee of Sun Yat-Sen University and housed in barrier facilities on a 12 h light/dark cycle. BALB/c-nu mice (4-5 weeks of age, 18-20 g) were purchased from the Center of Experimental Animal of Guangzhou University of Chinese Medicine. The BALB/c nude mice were randomly divided into groups (n = 6/group). One group of mice was inoculated subcutaneously with HepG2/Vector cells (5× 10^6^) in the left dorsal flank and with HepG2/mir-432 cells (5× 10^6^) in the right abdomen flank per mouse. Another two groups were inoculated subcutaneously per mouse with HepG2 cells (5× 10^6^) in the left abdomen flank. 7 days later, the mice were then intratumorally injected with one hundred microliters of antagomir control or antagomir-432 (diluted in PBS at 2 mg/ml) 3 times per week for 2 weeks. Tumors were examined twice weekly; length, width, and thickness measurements were obtained with calipers and tumor volumes were calculated. Tumor volume was calculated using the equation *volume (mm^3^)* = (L × W^2^)/2. On day 40, tumors were detected by an IVIS imagining system (Caliper), then animals were euthanized, tumors were excised, weighed and paraffin-embedded. Serial 6.0μm sections were cut, deparaffinized and subjected to IHC using an anti-Ki67 (Dako, Glostrup, Denmark) to provide histological evidence of the tumor phenotype. The proliferation index was quantified by counting the proportion of Ki67-positive cells

### Statistical analysis

All data were expressed as the mean ± SD. The Student's t-test was used to evaluate the statistical significance of differences between two groups of data in all pertinent experiments. *P* < 0.05 (using a two-tailed paired t-test) was thought to be significantly different for two groups of data.

## SUPPLEMENTARY MATERIAL FIGURES


