# Supplementary material for: Downregulation of miR-432 activates Wnt/β-catenin signaling and promotes human hepatocellular carcinoma proliferation
Source: Oncotarget. 2015 Mar 8;6(10):7866–79. doi: 10.18632/oncotarget.3492 (PMC4480722; doi:10.18632/oncotarget.3492)
Supplement: Supplementary file 1 [file oncotarget-06-7866-s001.pdf]

Downregulation of miR-432 activates Wnt/β-catenin signaling and promotes human hepatocellular carcinoma proliferation

Supplementary Material

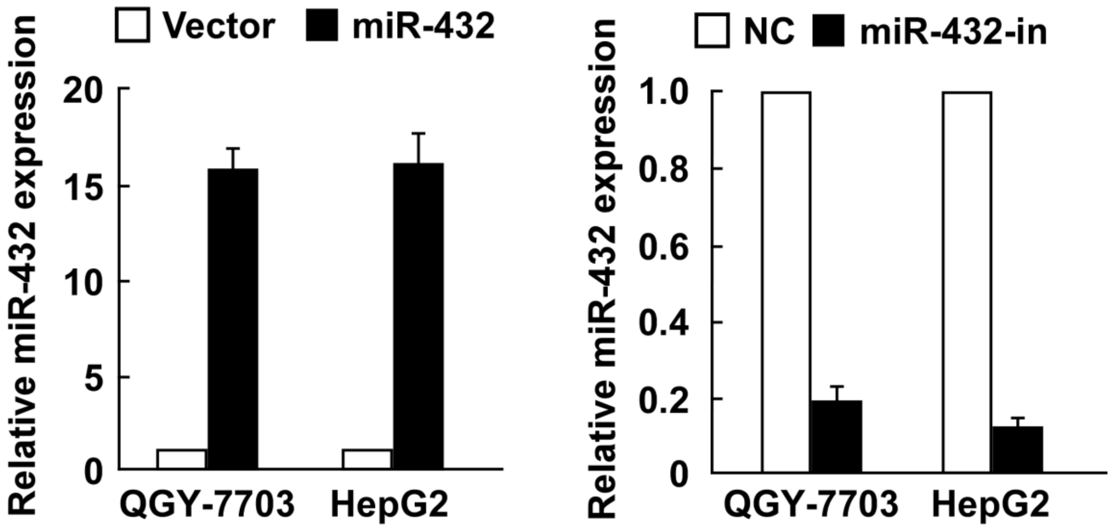

Supplementary Fig. 1: Real-time PCR analysis of miR-432 in miR-432 overexpressing and silencing cells. Transcript levels were normalized by *U6* expression. Error bars represent the mean ± SD from three independent experiments. * $P < 0.05$.

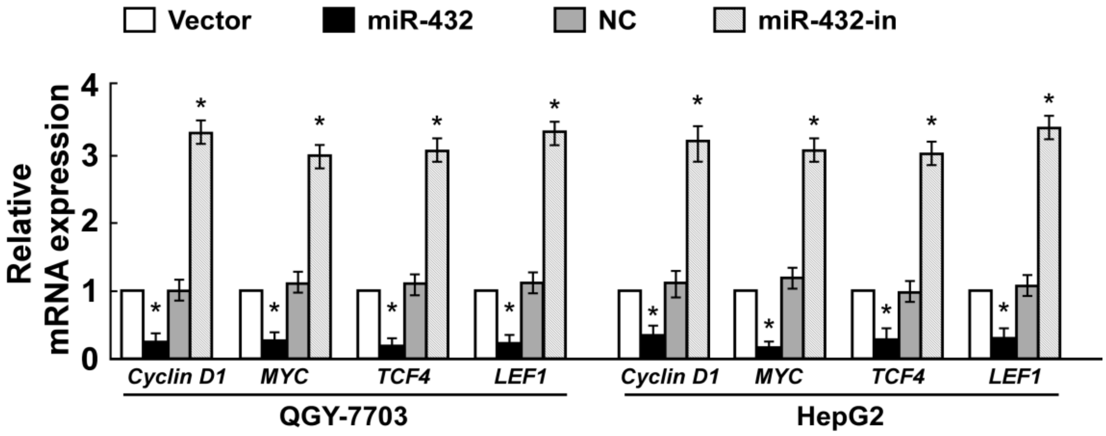

Supplementary Fig. 2: Real-time PCR analysis of mRNA expression of Cyclin D1, MYC, TCF4 and LEF1 in indicated HCC cells. Each bar represents the mean ± SD of three independent experiments. *$P < 0.05$.

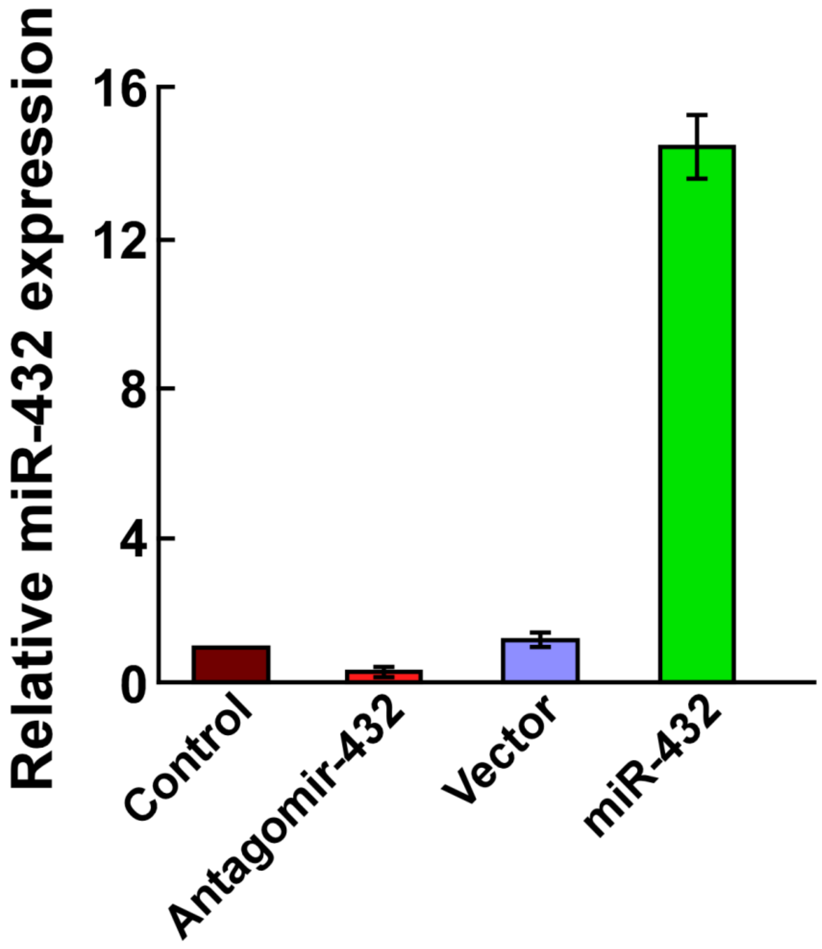

Supplementary Fig. 3: Real-time PCR analysis of miR-432 in the indicated xenografts tumors. *$P < 0.05$.

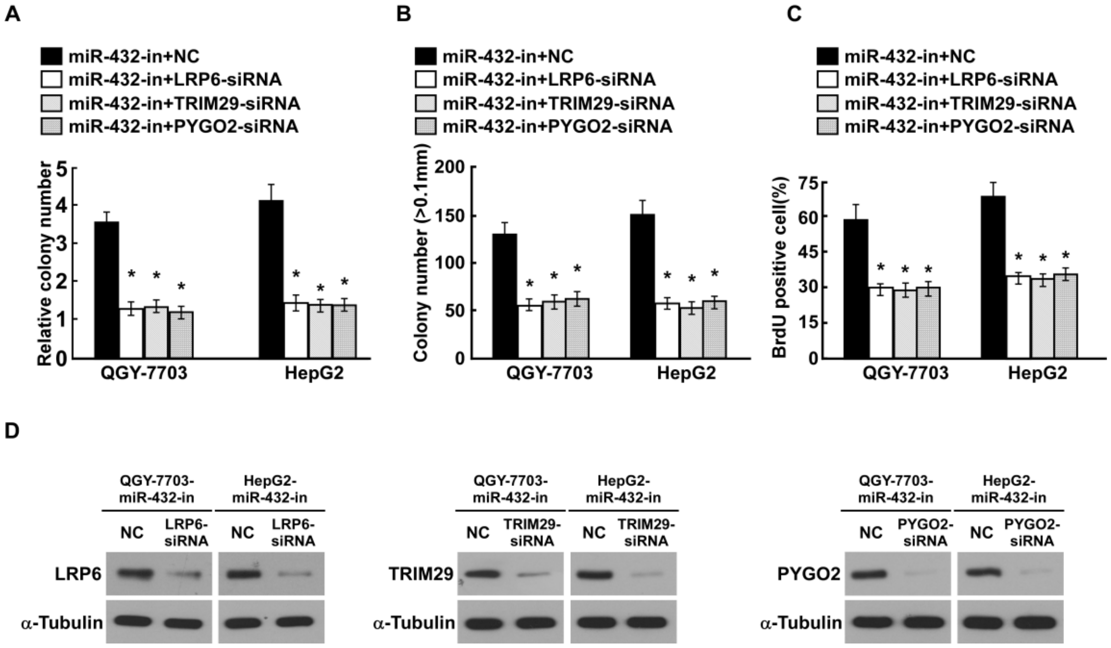

Supplementary Fig. 4: Suppression of LRP6, TRIM29, and Pygo2 is functionally important for the biological effects of miR-432 in HCC. (A). Quantification of crystal violet stained HCC cell colonies formed, 10 days after inoculation. (B). Quantification of colony numbers as determined by anchorage-independent growth assay. Colonies larger than 0.1 mm in diameter were scored. (C). Quantification of BrdU positive signaling in the cells. (D). Western blotting analysis of the expression levels of LRP6,TRIM29 and Pygo2 expression in miR-432-in transfected HCC cells which transfected with LRP6-siRNA, TRIM29-siRNA, or Pygo2-siRNA.; α-Tubulin served as loading control. Each bar represents the mean ± SD of three independent experiments. *$P < 0.05$.
